# The American Cyclopædia of Practical Medicine and Surgery

**Published:** 1837

**Authors:** 


### Art. VIII.—The American Cyclopædia of Practical Medicine and Surgery.

We have already noticed this valuable publication. Not having lately received a number, we wrote to the enterprising Editor, Dr. Hays, and learned, with regret, that its publication has, for the present, been suspended for want of patronage! Completed, this work would, of itself, be a respectable medical library; and being compiled by physicians of our own country, its precepts would have an applicability to the diseases we are called upon to treat, which could not be found in foreign digests, however learned. Why then should it be suffered to languish—perhaps expire? Our brethren have certainly not opened their eyes to its value, and to the duty of encouraging a work, which, finished in the manner it has been begun, would do credit to the American profession. Indeed, it is the only kind of publication, that can present to the old world, the discoveries and improvements of the new; and every American physician, therefore, has an interest in its completion. We respectfully call upon our brethren of the periodical press, to unite in our humble endeavor to arouse the practitioners of the United States, to an efficient encouragement of this undertaking; the failure of which, may discourage any similar work for many years to come. D.

